# Salvianolic Acid B Inhibits ERK and p38 MAPK Signaling in TGF-**β**1-Stimulated Human Hepatic Stellate Cell Line (LX-2) via Distinct Pathways

**DOI:** 10.1155/2012/960128

**Published:** 2011-08-11

**Authors:** Zhigang Lv, Lieming Xu

**Affiliations:** ^1^Shuguang Hospital, Shanghai University of Traditional Chinese Medicine, Shanghai 201203, China; ^2^Institute of Liver Diseases, Shanghai University of Traditional Chinese Medicine, Shanghai 201203, China; ^3^Department of Neurology, Memorial Sloan-Kettering Cancer Center, New York, NY 10021, USA; ^4^Key Laboratory of Liver and Kidney Diseases, Shanghai University of Traditional Chinese Medicine, Ministry of Education, Shanghai 200444, China; ^5^E-Institute of Traditional Chinese Medicine Internal Medicine in Shanghai University, Shanghai 201203, China

## Abstract

Salvianolic acid B (SA-B) is water-soluble component of *Radix Salvia miltiorrhiza*. The previous work indicated that SA-B can inhibit MAPK and Smad signaling in activated hepatic stellate cells (HSCs) to perform anti-fibrotic activity Lv et al. 2010. However, some studies have shown that there is cross-talk between MAPK and Smad in certain cell types. Thus, the anti-fibrotic action of SA-B may be through the cross-talk. In order to clarify the mechanism of SA-B further, we knocked down Smad in LX-2 cells (SRV4) via RNAi, and then added TGF-**β**1, and PD98059 or SB203580 and SA-B. The levels of p-MEK and p-p38 were inhibited by SA-B in SRV4 independent of TGF-**β**1. The expression of Col I and **α**-SMA in SRV4 could be reduced by SA-B independent TGF-**β**1. SB203580 had not significant effect on p-MEK in SRV4 stimulated by TGF-**β**1. The levels of p-MEK in SRV4 were not increased significantly after TGF-**β**1 stimulation. PD98059 had no effect on the levels of p-p38 in SRV4 irrespective of TGF-**β**1. In conclusion, SA-B inhibits the synthesis of Col I in LX-2 cells independent of TGF-**β**1 stimulation, and the anti-fibrotic effect of SA-B is due to direct inhibition of p38 signaling and inhibition the cross-talk of Smad to ERK signaling.

## 1. Introduction

Hepatic fibrosis is a wound-healing response to chronic liver injury. It is a scarring process associated with an increased deposition of fibrillar extracellular matrix in liver [1]. Much progress has been made in understanding the cellular mechanisms of hepatic fibrosis. Activation of HSCs is the central event in this condition [2]. Following liver injury of any etiology, HSCs undergo a transition from quiescent vitamin A-rich cells into proliferative, fibrogenic, and contractile myofibroblasts, in which synthesis of ECM and particularly Col. I predominates [3]. 

Transforming growth factor *β*1 (TGF-*β*1) is one of the most powerful and widely distributed profibrogenic mediators. It is the dominant stimulator of ECM production by HSCs, regulating deposition of the ECM as part of the normal response to tissue injury and pathological fibrosis [4, 5]. Alterations in TGF-homeostasis are important factors in fibrotic diseases of multiple tissues. The pathogenic relevance of TGF-*β*1 to hepatic fibrosis has been established and the signaling pathway induced by TGF-*β*1 in HSCs is fully understood. It is clear that MAPK and Smad signaling pathways in HSCs activated by TGF-*β*1 lead to excess ECM deposition. However, up to now, there are not effective measures to stop or reverse the disease progression. 

In recent years, increasing attention has been directed to the anti-fibrotic activity of natural herbs, in particular *Salviae miltiorrhiza*, a traditional Chinese herbal medicine widely used for anti-fibrosis in clinics. In order to explore the mechanism of action of *Salvia miltiorrhiza*, Salvianolic acids B (SA-B), an effective water-soluble component of *Radix Salvia miltiorrhiza*, was investigated. Previous research indicated that SA-B could effectively reverse liver fibrosis in patients suffering from chronic hepatitis B, and it was more effective than IFN-*γ* in reducing serum HA, PC-III, Col. IV, and LM contents and decreasing ultrasound fibrosis imaging scores [6]. SA-B inhibits TGF-*β*1 secretion in activated HSCs and inhibits the expression of Col. I stimulated by TGF-*β*1. SA-B inhibits the Smad and MAPK pathway in HSCs stimulated with TGF-*β*1 [7, 8].

 It has been reported that a synergy between TGF-*β*1 activated extracellular signal-regulated kinase (ERK) and Smad signaling in collagen production by human glomerular mesangial cells, and dominant negative ERK blockade reduced TGF-*β*1 induced phosphorylation of Smad2/3. This effect was not seen in the mouse mammary epithelial NMuMG cell line, indicating that ERK-dependent activation of Smad2/3 occurs only in certain cell types [9, 10]. These results indicate that ERK-dependent R-Smad (receptor-regulated Smad) linker region phosphorylation enhances collagen I synthesis and imply positive cross-talk between the ERK and Smad pathways in human mesangial cells. However, it is not clear if there is a cross-talk between MAPK and Smad signaling pathways in TGF-*β*1-stimulated HSCs. Thus, in order to clarify the targets of SA-B on anti-fibrotic action, the cross-talk would have to be inhibited. 

This study investigated the anti-fibrotic role of SA-B in HSCs via inhibiting the Smad and MAPK signaling pathways or by inhibiting the cross-talk between them.

## 2. Materials and Methods

### 2.1. Reagents

 Smad4 polyclonal antibody, p-p38 monoclonal antibody, p38, p-MEK and MEK monoclonal antibody were purchased from Cell Signaling Technology (Beverly, MA, USA); mouse anti-human *α*-SMA monoclonal antibody and Trizol reagent were purchased from Sigma (St Louis, MO, USA). BLOCK-iT Pol II miR RNAi expression vector was purchased from Invitrogen (Carlsbad, CA, USA). Rabbit anti-human type I collagen antibody, SB203580 (p38 signaling inhibitor) and PD98059 (ERK signaling inhibitor) were purchased from Chemicon (Temecula, CA, USA). Mouse anti-GAPDH monoclonal antibody was purchased from Sigma (St Louis, MO, USA) and TGF-*β*1 was supplied by R&D Systems (Minneapolis, MN, USA). The human Type I Collagen Detection Kit was purchased from Chondrex (Redmond, WA) and SA-B (purity > 98%; molecular formula: C_36_H_30_O_16_; molecular weight: 718.62) was kindly provided by Dr. Zhu Dayuan(Shanghai Institute of Materia Medica, Shanghai, China). SA-B powder was stored at 4°C. SA-B was dissolved before use.

### 2.2. RNA Silencing and Cell Transfection

 RNA silencing was performed using the BLOCK-iT Pol II RNAi expression vector kit following the manufacturer's protocol. Artificial Smad4 microRNA (miRNA) was cloned in pcDNA 6.2-GW/EmGFP-miR leading to cocistronic expression of Emerald GFP (EmGFP) with miRNA of Smad4. Different sequences of Smad4 miRNA were designed using an algorithm developed by Invitrogen. Sense and antisense DNA sequences were *SR1-Forward: TGC TGT ATG ATG GTA AGT AGC TGG CTG TTT TGG CCA CTG ACT GAC AG CCA GCT TTA CCA TCA TA *and *SR1-Reverse*: *CCT GTA TGA TGG TAA AGC TGG CTG TCA GTC AGT GGC CAA AAC AGC CAG CTA CTT ACC ATC ATA C*; *SR2-Forward: TGC TGT GGT GAG GCA AAT TAG GTG TGG TTT TGG CCA CTG ACT GAC CAC ACC TAT TGC CTC ACC A* and *SR2-Reverse: CCT GTG GTG AGG CAA TAG GTG TGG TCA GTC AGT GGC CAA AAC CAC ACC TAA TTT GCC TCA CCA C; SR3-Forward*:* TGC TGT TTC CGA CCA GCC ACC TGA AGG TTT TGG CCA CTG ACT GAC CTT CAG GTC TGG TCG GAA A; SR3-Reverse*:* CCT GTT TCC GAC CAG ACC TGA AGG TCA GTC AGT GGC CAA AAC CTT CAG GTG GCT GGT CGG AAA C; SR4-Forward*: *TGC TGA TTA CTT GGT GGA TGC TGG ATG TTT TGG CCA CTG ACT GAC ATC CAG CAC ACC AAG TAA T,* and *SR4-Reverse*: *CCT GAT TAC TTG GTG TGC TGG ATG TGT CAG TCA GTG GCC AAA ACA TCC AGC ATC CAC CAA GTA ATC*. LX-2 cells [11], human HSCs, supplied by Scott L. Friedman, were cultured in DMEM medium supplemented with 10% fetal bovine serum at 37°C in 5% CO_2_ and were transfected with 10 *μ*g of the indicated vectors. Transfected cells were selected by 5 mg/mL blasticidin.

### 2.3. RT-PCR and Quantitative PCR Analysis of Gene Expression

 Total RNA was extracted from selected LX-2 cells using Trizol reagent, quantified by spectrophotometry, and reverse-transcribed to cDNA using the Invitrogen Reverse Transcription System. An iCycleriQ Multicolor Real time PCR (RT-PCR) Detection System (Bio-Rad) was used for RT-PCR. The cDNA of LX-2 was amplified using iQ-SYBR Green Supermix with specific oligonucleotide primers for Smad4 and *β*-actin (for normalization). Gene primers were Smad4 *Forward: *5′*-ATT GCC GAC AGG ATG CAG A-*3′*; Reverse: *5′*-GAG TAC TTG CGC TCA GGA GGA-*3′*; β*-actin *Forward: *5′*-TCT CTC AAT GGT TTC TGT CCT GTG-*3′*; Reverse: *5′*-TCC CAT TTC CAA TCA TCC TGC TC-*3′. Threshold cycles (Ct) were automatically calculated by the RT-PCR System. Each Ct value was normalized to the *β*-actin Ct value. Relative quantification was expressed as fold-induction compared to control conditions. PCR amplification was performed in a 50 *μ*L reaction mixture containing 1 × PCR amplification buffer, 1.5–2 mM MgCl_2_ (optimized for each reaction), 25 pmol of each primer, 0.2 mM dNTPs and 2.5 units of AmpliTaq DNA polymerase (Perkin Elmer Applied Biosystem). The PCR program was as follows: 95°C for 5 min, 35 cycles of 2 min at 94°C, 1.5 min at 60°C, 2.5 min at 72°C, and a final extension for 10 seconds at 72°C. The PCR products were separated on a 2% agarose gel stained with ethidium bromide and compared to a standard DNA size marker. 

### 2.4. Treatment of LX-2 Cells and Preparation of Cell Lysates

 LX-2 cells transfected with SRV4 expressing high levels of Smad4 RNAi, and a negative control vector, were selected by blasticidin and cultured in DMEM medium supplemented with 10% FSC. SA-B (10^−6^ M L^−1^) (The concentration is near to clinical application dose.) was added to LX-2 cells in serum-free medium for 2 days. TGF-*β*1 (10 ng mL^−1^) was added to the medium during the last 2 hours of culture, or for the last 24 h when carrying out gene transfection examinations. Specific protein kinase blocker (50 *μ*M), PD98059 (blocking ERK signaling) or SB203580 (blocking p38 MAPK signaling), was added to the media 30 min before the addition of TGF-*β*1. After TGF-*β*1 stimulation, LX-2 cells were washed with PBS and lysed in 50 M Tris-HCl, pH 7.5, 150 mM NaCl, 1% Triton X-100, 0.1 mM Na_3_VO_4_, 1 mM PMSF, and 0.1 mM aprotinin. Cell debris was removed by centrifugation at 15000× g for 30 min at 4°C. Protein concentrations were determined using BioRad protein assay.

### 2.5. Western Blotting

 Samples were subjected to 10% sodium dodecyl sulphate-polyacrylamide gel electrophoresis and transferred to nitrocellulose membranes. Membranes were blocked and washed in TBS-Tween buffer. They were incubated overnight with specific antibodies (Smad4, *β*-Actin, MEK, p38, p-p38, GAPDH, *α*-SMA or Col. I). After incubation with peroxidase-conjugated secondary antibodies for 1 h, membranes were developed with an enhanced chemiluminescence western blotting detection system. The experiment was repeated for three times.

### 2.6. Detection of Collagen I in Media Using ELISA

 Cell culture media were centrifuged and supernatants collected for measuring total protein concentrations: 90 *μ*L sample and 10 *μ*L 10 × coating buffer (1.59% Na_2_CO_3_, 2.93% NaHCO_3_, pH 9.6) were added to wells of a 96-well plate in triplicate; Col. I standard controls were added to standard wells [1 × coating buffer (0.159% Na_2_CO_3_, 0.293% NaHCO_3_, pH 9.6)]. The 96-well plate was incubated at 4°C and coated for 24 h. The coating buffer was discarded and the plate washed with washing buffer three times for 2 minutes. Rabbit anti-mouse Col. I (1 : 200) diluted with 0.01 M PBS was added to each well in a volume of 100 *μ*L and incubated at 37°C for 2 h. Goat anti-rabbit IgG-HRP diluted (1 : 1000) with enzyme-labeled antibody dilution (90% 0.01 M PBS, 10% inactivated bovine serum) was added to each well in a 100 *μ*L volume and plates were incubated at 37°C for 2 h. The preparations were developed under darkroom conditions at room temperature. After the chromatism of the standard well was observable, 2 N H_2_SO_4_ (100 *μ*L/well) was added to stop the reaction. The absorbance was read at 492 nm on a microplate reader, and a standard curve was generated. The Col. I contents detected (ng mL^−1^) per total protein in the supernatant (mg mL^−1^) were recorded.

### 2.7. Data Analysis

Data are expressed as mean ± standard deviation. Data were analyzed using a one-way analysis of variance as well as the least significant difference test, and *P* < 0.05 was considered statistically significant. 

## 3. Results

### 3.1. Construction of LX-2 Cell-Line Containing Smad4 RNAi

**Figure 1:**
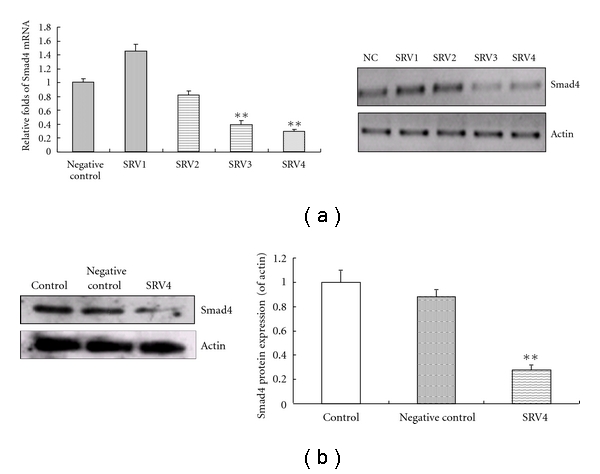
Inhibition of Smad4 in LX-2 transfected with Pol II miR RNAi expression vectors (SRV1, SRV2, SRV3, and SRV4). (a) Smad4 mRNA expression levels detected by real time PCR 72 h after selection with blasticidin. Results were normalized to Smad4 expression in “Negative control” using the 2^−ΔΔCt^ method (where Ct is threshold cycle). **Significant difference versus negative control (*n* = 3, *P* < 0.01). On the right side of the figure is shown the electrophoresis of PCR product. (b) Smad4 protein expression levels detected after selection with blasticidin, using the Western blot. Blotting with anti-*β*-actin antibody was conducted as a protein loading control. Quantification of the intensity of bands calibrated to the intensity of total protein bands (means ± SD). **Significant difference versus negative control (*n* = 3, *P* < 0.01).

 The Pol II miR RNAi expression vector was constructed and confirmed by sequencing. The level of Smad4 mRNA in LX-2 cells transfected with SRV3 and SRV4 was decreased compared to control cells. After selection of cells with blasticidin, the protein levels of Smad4 in LX-2 cells transfected with SRV4 were reduced to approximately 30% (Figures 1(a) and 1(b)). The levels of Smad4 mRNA were reduced to approximately 70.3% compared to control (Figure 1(b)). 

### 3.2. The Effect of SA-B on p38 MAPK Signaling in LX-2 Cells Is via Inhibiting the Smad and ERK Pathways

**Figure 2:**
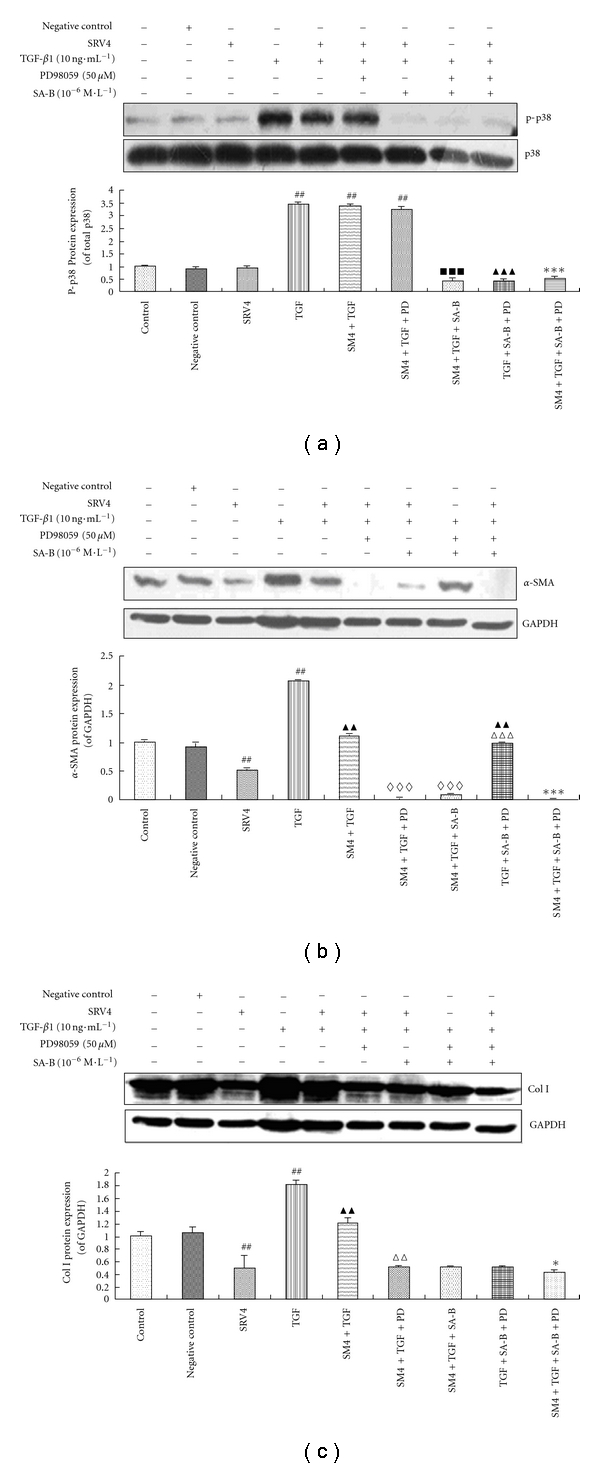
The effects of SA-B on p38 MAPK pathway via Inhibition of ERK and Smad signaling. (a) P38 phosphorylation in LX-2 cells. The levels of phosphorylated p38 protein were determined by Western blot using anti-phospho-p38 antibodies. The levels of total p38 protein were determined by Western blot using anti-p38 antibodies. Quantification of the intensity of bands calibrated to the intensity of total protein bands (means ± SD). ^##^Significant difference versus Control, Negative control, and SRV4 (*n* = 3, *P* < 0.01); ^■■■^Significant difference versus SM4 + TGF (*n* = 3, *P* < 0.001); ^▲▲▲^Significant difference versus TGF (*n* = 3, *P* < 0.001); ***Significant difference versus SM4 + TGF + PD (*n* = 3, *P* < 0.001). (b) *α*-SMA level in LX-2. The levels of *α*-SMA protein were determined by Western blot using anti-*α*-SMA antibodies. Blotting with anti-GAPDH antibodies was conducted as a protein loading control. Quantification of the intensity of bands calibrated to the intensity of total protein bands (means ± SD). ^##^Significant difference versus Control, Negative control, and SRV4 (*n* = 3, *P* < 0.01); ^▲▲^Significant difference versus TGF (*n* = 3, *P* < 0.01); ^*⋄⋄⋄*^Significant difference versus SM4 + TGF (*n* = 3, *P* < 0.001), ^*▵▵▵*^Significant difference SM4 + TGF + PD and SM4 + TGF + SA-B (*n* = 3, *P* < 0.001); ***Significant difference versus SM4 + TGF + SA-B, TGF + SA-B + PD (*n* = 3, *P* < 0.001). (c) Col. I level in LX-2. The levels of Col. I protein were determined by Western blot using anti-Col. I antibodies. Blotting with anti-GAPDH antibodies was conducted as a protein loading control. ^##^Significant difference versus Control, Negative control, and SRV4 (*n* = 3, *P* < 0.01); ^▲▲^Significant difference versus TGF (*n* = 3, *P* < 0.01); ^*▵▵*^Significant difference versus SM4 + TGF (*n* = 3, *P* < 0.01); *Significant difference versus SM4 + TGF + PD, SM4 + TGF + SA-B, and TGF + SA-B + PD (*n* = 3, *P* < 0.05).

 Without TGF-*β*1 stimulation and SA-B drug intervention, p-p38 protein can be detected in LX-2 cells. When LX-2 cells were transfected with a negative control plasmid or a SRV4 vector, there was no significant effect on p-p38 protein expression, however, following to stimulation with TGF-*β*1 the levels of p-p38 protein were significantly increased (*P* < 0.01). The LX-2 cells containing Smad4 RNAi or Smad4 RNAi combined with PD98059 expressed higher levels of p-p38 protein when they were stimulated with TGF-*β*1 (*P* < 0.01). In the case of LX-2 cells containing Smad4 RNAi, the p-p38 protein expression levels in LX-2 cells stimulated with TGF-*β*1 were significantly inhibited by SA-B (*P* < 0.001 in all cases) (Figure 2(a)). Similar effect was observed when LX-2 cells containing Smad4 RNAi combined with PD98059 addition (*P* < 0.001 in all cases) (Figure 2(a)).

 We determined the changes of *α*-SMA and Col. I, which are markers of activated HSCs, following inhibition of the Smad and ERK pathways. The protein expression of *α*-SMA and Col. I was high in control cells and the cells transfected with a negative control vector, but the levels of protein were much lower in the SRV4 group. TGF-*β*1 stimulation of LX-2 cells increased the protein expression of *α*-SMA and Col. I markedly (*P* < 0.01), but the levels were significantly lower in the SRV4 + TGF group (*P* < 0.01) (Figures 2(b) and 2(c)). The Col. I protein content in the supernatants from these groups showed similar trends (Table 1). In LX-2 cells containing Smad4 RNAi combined with PD98059 addition and TGF stimulation, the protein expression of *α*-SMA was virtually undetectable. In LX-2 cells containing Smad4 RNAi, combined with TGF-*β*1 stimulation, the expression of *α*-SMA and Col. I was significantly inhibited by SA-B or PD98059 (Figure 2(c)).

### 3.3. The Effect of SA-B on ERK Signaling in LX-2 Cells Is via Inhibiting the Smad and p38 Signaling

**Figure 3:**
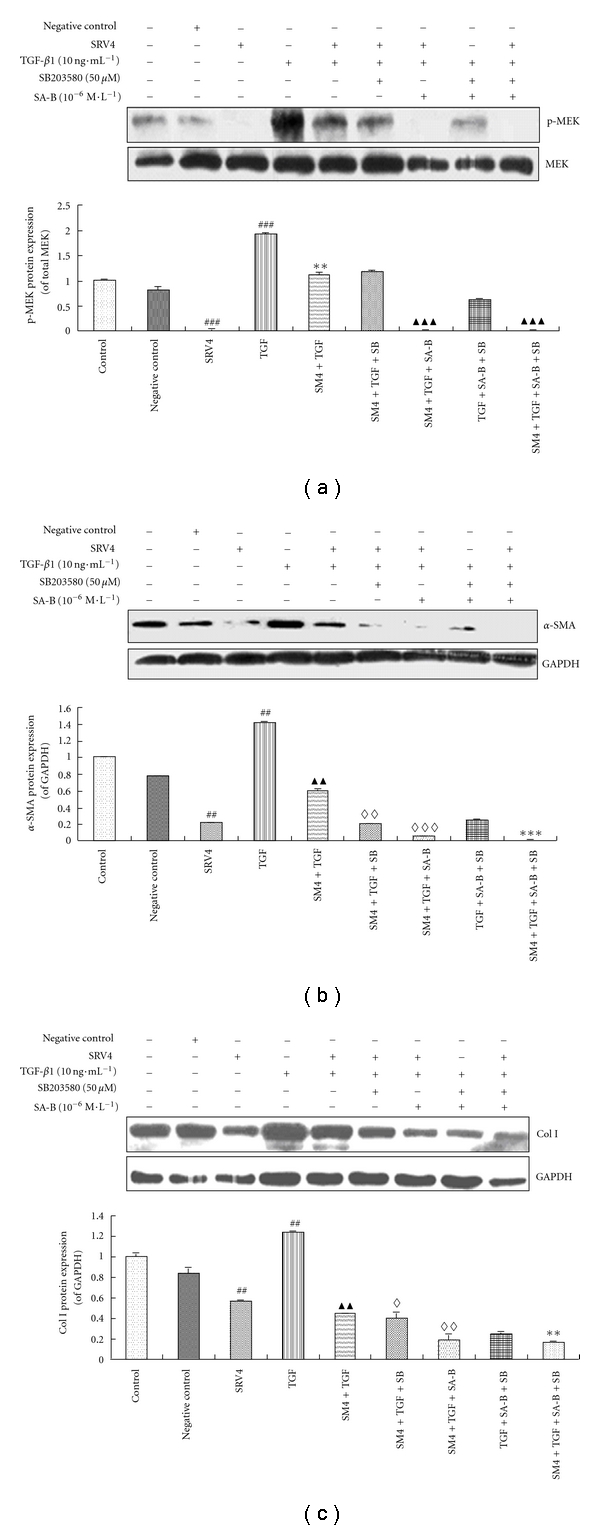
The effects of SA-B on ERK signaling via inhibition of p38 MAPK and Smad signaling. (a) MEK phosphorylation in LX-2 cells. The levels of phosphorylated MEK protein were determined by Western blot using anti-phospho-MEK antibodies. The levels of total MEK protein were determined by Western blot using anti-MEK antibodies. Quantification of the intensity of bands calibrated to the intensity of total protein bands (means ± SD). ^###^Significant difference versus Control, Negative control, and SRV4 (*n* = 3, *P* < 0.001); **Significant difference versus TGF (*n* = 3, *P* < 0.01); ^▲▲▲^Significant difference versus SM4 + TGF, SM4 + TGF + SB, and TGF + SA-B + SB (*n* = 3, *P* < 0.01). (b) *α*-SMA levels in LX-2. The levels of *α*-SMA protein were determined using anti-*α*-SMA antibodies. Blotting with anti-GAPDH antibodies was conducted as a protein loading control. ^##^Significant difference versus Control, Negative control and SRV4 (*n* = 3, *P* < 0.01); ^▲▲^Significant difference versus TGF (*n* = 3, *P* < 0.01); ^*⋄⋄*^Significant difference versus SM4 + TGF (*n* = 3, *P* < 0.01); Significant difference versus SM4 + TGF (*n* = 3, *P* < 0.001); ***Significant difference versus SM4 + TGF + SB, SM4 + TGF + SA-B, and TGF + SA-B + SB (*n* = 3, *P* < 0.001). (c) Col. I level in LX-2. The levels of Col. I protein were determined by Western blot using anti-Col. I antibodies. Blotting with anti-GAPDH antibodies was conducted as a protein loading control. ^##^Significant difference versus Control, Negative control, and SRV4 (*n* = 3, *P* < 0.01); ^▲▲^Significant difference versus TGF (*n* = 3, *P* < 0.01); ^*◊*^Significant difference versus SM4 + TGF (*n* = 3, *P* < 0.05); ^*◊◊*^Significant difference versus SM4 + TGF (*n* = 3, *P* < 0.01); **Significant difference versus SM4 + TGF + SB, SM4 + TGF + SA-B, and TGF + SA-B + SB (*n* = 3, *P* < 0.01).

 Our previous research results indicate that SA-B can inhibit the expression of p-MEK, but have no significant effect on other kinases of ERK pathway [12]. p-MEK was detected in LX-2 cells in the absence of TGF-*β*1 stimulation and SA-B intervention. p-MEK level was significantly increased by TGF-*β*1 stimulation (*P* < 0.001). When Smad signaling was knocked down, p-MEK expression was significantly decreased irrespective of TGF-*β*1 stimulation (*P* < 0.01), and there was no statistically significant difference in p-MEK protein expression in the SRV4 + TGF + SB group or the SRV4 + TGF group. Furthermore, p-MEK protein was virtually undetectable in the SRV4 + TGF + SA-B and SRV4 + TGF + SA-B + SB groups (*P* < 0.001) (Figure 3(a)). 

 Similar to 3.2, protein expression of *α*-SMA and Col. I was detectable in control cells and those transfected with a negative control vector, but it was significantly lower in the SRV4 group (*P* < 0.01). LX-2 cells stimulated by TGF-*β*1 had significantly increased *α*-SMA and Col. I expression (*P* < 0.01); and significantly reduced *α*-SMA and Col. I in the SRV4 + TGF group (*P* < 0.001). LX-2 cells in the SRV4 + TGF + SB group expressed a low amount of *α*-SMA (*P* < 0.01). In the SRV4 + TGF + SA-B group and SRV4 + TGF + SA-B + SB group, the *α*-SMA level was minimal. The protein expression of *α*-SMA in LX-2 cells of the TGF + SA-B + SB group was significantly lower than that of the TGF + SA-B group and TGF + SB group (both *P* < 0.01) (Figure 3(b)).The level of Col. I in the SRV4 + TGF + SB group and SRV4 + TGF + SA-B group was lower than in the SRV4 + TGF group (both *P* < 0.05). In the SRV4 + TGF + SA-B + SB group, the protein expression of Col. I was lower than in the SRV4 + TGF + SA-B and TGF + SA-B + SB groups (both *P* < 0.01) (Figure 3(c)). Similar changes of Col. I can be also observed in culture media of LX-2 (Table 2). 

## 4. Discussion

 General TGF-*β*1 signaling pathways have been described in detail. In addition to the Smad signaling pathway, TGF-*β*1 can persistently activate MAPK signaling pathways [13, 14]. A variety of extracellular stimuli transmit signals from cell membrane to nucleus and converge on MAPK cascades [15]. Three distinct MAPK pathways have been described in mammalian cells, the extracellular signal-regulated kinases (ERK) pathway, the c-Jun amino terminal kinase (JNK) pathway, and the p38 MAPK pathway [16]. TGF-*β*1 activates ERK and p38MAPK signaling in HSCs [17, 18].

SA-B inhibits Smads and MAPK activity in HSCs stimulated with TGF-*β*1 [8, 19]. However, the mechanism(s) by which SA-B regulates the MAPK signaling pathway in HSCs stimulated with TGF-*β*1 have not been elucidated completely.

In the present study, we used RNAi to knock down Smad4 protein expression, thus inhibiting potential cross-talk of the Smads to the MAPK signaling. The BLOCK-iT Pol II miR RNAi expression vector system was used to knock down Smad4 mRNA levels. Smad4 mRNA levels were decreased by 70.3% in LX-2 cells selected with blasticidin. This allowed us to investigate the anti-fibrotic action of SA-B in depth.

### 4.1. Cross-Talk between the Smad and MAPK Signaling Pathways

**Figure 4:**
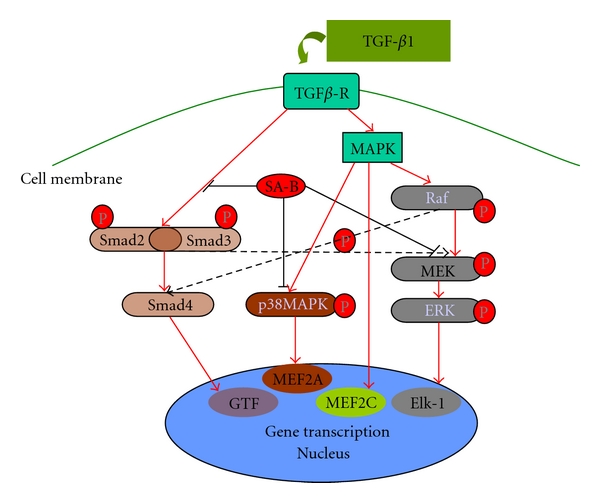
Proposed mechanisms of the anti-fibrotic effects of SA-B on activated HSCs stimulated by TGF-*β*1.

 TGF-*β*1 stimulates further activation of LX-2 cells. Furthermore, phosphorylation of p38 and ERK was significantly increased. In LX-2 cells affected by Smad4 RNAi, the phosphorylation of p38 was not significantly different compared to control. However, the protein expression of p-p38 and p-ERK in LX-2 cells stimulated with TGF-*β*1 was significantly higher than in controls. Smad4 RNAi did not inhibit the phosphorylation of p38 in LX-2 cells stimulated with TGF-*β*1. This suggests there may be lack of “cross-talk” of Smad signaling pathway to the p38 MAPK signaling pathway in LX-2 cells stimulated with TGF-*β*1. In the Smad4 RNAi LX-2 cells with ERK pathway inhibitor, TGF-*β*1 induced increased expression of p-p38. This indicates that there seems to be no “cross-talk” between the ERK signaling pathway and p38 signaling pathway in HSCs. In Smad4 RNAi LX-2 cells stimulated with TGF-*β*1, the protein expression of p-MEK was significantly lower compared to that in the cells without Smad4 RNAi. These results indicate there may be “cross-talk” between the Smad pathway and the ERK pathway (Figure 4). Such cross-talk has been reported in other types of cells [20–22]. In Smad4 RNAi LX-2 cells stimulated with TGF-*β*1, SB203580 did not have a significant effect on the expression of p-MEK. This suggests there may be no significant cross-talk between the p38 pathway and the ERK pathway (Figure 4). However, cross-talk between ERK and p38 MAPK has been reported in other cell types [23]. This indicates the complexity of cross-talk and signaling pathways in the different cell types.

### 4.2. The Inhibitory Effect of SA-B on ERK Signaling in HSCs Stimulated with TGF-*β*1

 SA-B inhibits the phosphorylation of MEK in HSCs, but has no significant effect on phosphorylation of other kinases in ERK signaling. SA-B also has no significant effect on total MEK protein expression [12]. In view of the potential “cross-talk” between the Smad and the ERK signaling pathway, further clarification of the effect of SA-B on the ERK signaling pathway was required. To investigate this, the Smad signaling pathway was inhibited and the effect of SA-B on the phosphorylation of MEK was analyzed.

As shown in Figure 3(a), SA-B suppresses the phosphorylation of MEK significantly. In Smad4 RNAi HSC cells, SA-B inhibits MEK phosphorylation with or without the addition of SB203580. This indicates that there is no cross-talk between the p38 and ERK pathways. The phosphorylation level of MEK in the SRV4 + TGF group was 50% of that in the TGF group, and after Smad4 knockdown combined with SA-B intervention, the phosphorylation level of MEK was barely detectable by Western blotting. The decline in phosphorylation levels of MEK in HSCs may not only be due to the residual levels of Smad4 but also to the action of SA-B. Therefore, it may be concluded that SA-B directly inhibits the phosphorylation of MEK in HSCs (Figure 4).

### 4.3. The Inhibitory Effect of SA-B on the p38 Signaling Pathway in HSCs Stimulated by TGF-*β*1

MAPK signaling has been implicated in TGF-*β*1-mediated signaling and collagen gene expression [15]. In MAPK family, ERK and p38 MAPK activation can increase the collagen in hepatic stellate cells. The data shown in Figures 2(a) and 3(a) illustrate the lack of cross-talk between p38 MAPK and the Smad or ERK signaling pathways. The reduction of phosphorylated p38 may be due to SA-B. In previous research we found SA-B has no significant effect on total MEK protein expression [12].

Following Smad4 RNAi and/or blocking ERK signaling, SA-B inhibits the expression of p-p38 in HSCs stimulated with TGF-*β*1. This confirms that SA-B inhibits the phosphorylation of p38 in HSCs independent of the Smad and ERK pathways (Figure 4). To clarify the action of SA-B on p38MAPK signaling pathway, it will be required to detect the kinase upstream of p38.

### 4.4. SA-B Suppresses the Expression of Col. I and *α*-SMA in HSCs Stimulated with TGF-*β*1 via Inhibition of MEK Phosphorylation in ERK Signaling and p38MAPK Signaling

 The protein expression of *α*-SMA can be detected in control cells. Smad4 RNAi caused a significant reduction in the expression of *α*-SMA irrespective of TGF-*β*1 stimulation. Smad4 RNAi resulted in further reduction in *α*-SMA expression with ERK or p38 MAPK signaling being inhibited. In the SRV4 + TGF + SA-B, SRV4 + TGF + SA-B + SB, and SRV4 + TGF + SA-B + PD groups, the protein expression of *α*-SMA could not be detected. 

In the SRV4 + TGF + SA-B, and SRV4 + TGF + PD groups, the expression of Col. I was inhibited by SA-B, an effect similar to PD98059. In these groups, the SA-B inhibitory action is weaker than that of Smad4 RNAi, compared to the TGF + SA-B + PD and SRV4 + TGF + SA-B + PD groups. As shown in Figure 3(c), SA-B suppresses the expression of Col. I more effectively than SB20358 in the SRV4 + TGF + SA-B and SRV4 + TGF + SB groups. However, in the TGF + SA-B + SB, SRV4 + TGF + SA-B + SB, SRV4 + TGF + SA-B and SRV4 + TGF + SB groups, we could not determine which was most effective on reducing the production of Col. I in HSCs in Smad4 RNAi, SB20358, or SA-B. 

SA-B has a significant inhibitory effect on Smad and MAPK signaling. The anti-fibrotic effect of SA-B occurs via inhibiting p38MAPK signaling pathway directly and inhibiting the cross-talk of Smad signaling pathway to ERK MAPK signaling pathway.

As demonstrated in Figures 2(c) and 3(c), when the ERK and p38 MAPK signaling pathways were blocked, respectively, Col. I expression was significantly inhibited. Smad4 RNAi enhanced the inhibitory effect on the intracellular expression of Col. I. This suggests that the Smad, ERK, and p38 pathways are involved in Col. I synthesis in activated HSCs. However, Smad4 RNAi (70.3% effective) and antagonists of ERK and p38 MAPK did not completely inhibit signal transduction in LX-2 cells stimulated with TGF-*β*1, and LX-2 cells express residual levels of Col. I protein. After weakening the Smad and MAPK signal pathways, the inhibitory action of SA-B on Col. I production is still evident. The expression of Col. I is the lowest in the SRV4 + TGF + PD + SB + SA-B group, but it can still be detected. A possible explanation for this result is that SA-B supplements the inhibitory action of Smad4 RNAi, PD98059, and SB203580 on the signal transduction pathway, but does not completely inhibit Col. I synthesis in HSCs stimulated with TGF-*β*1, suggesting the involvement of other signal transduction pathways. Normally cultured LX-2 cells express Col. I, suggesting that they are activated by mechanisms other than TGF-*β*1 stimulation, which requires further investigation. 

## Figures and Tables

**Table 1:** The changes of Col. I in LX-2 cell culture supernatants following inhibition of ERK and Smad4 signaling pathways.

Group	*n*	mean ± S
Control	3	0.567 ± 0.093
Negative control	3	0.469 ± 0.004
SRV4	3	0.351 ± 0.011^##^
TGF	3	1.007 ± 0.017^##^
SM4 + TGF	3	0.648 ± 0.012^▲▲^
SM4 + TGF + SA-B	3	0.289 ± 0.022
SM4 + TGF + PD	3	0.294 ± 0.023**
TGF + SA-B + PD	3	0.439 ± 0.003
SM4 + TGF + SA-B + PD	3	0.252 ± 0.004^♦♦^

The levels of Col. I protein were determined by ELISA kit as indicated. Results were expressed as mean ± SE of three independent experiments performed in triplicate. ^##^Significant difference versus Control, Negative control, and SRV4 (*n* = 3, *P* < 0.01); ^▲▲^Significant difference versus SRV4 and TGF (*n* = 3, *P* < 0.01); **Significant difference versus SM4 + TGF (*n* = 3, *P* < 0.01); ^♦♦^Significant difference versus SM4 + TGF + SA-B, SM4 + TGF + PD, and TGF + SA-B + PD (*n* = 3, *P* < 0.01).

**Table 2:** The changes of Col. I in LX-2 cell culture supernatant after inhibition of p38 MAPK and Smad4 signaling.

Group	*n*	Mean ± S
Control	3	0.567 ± 0.093
Negative control	3	0.469 ± 0.004
SRV4	3	0.351 ± 0.011
TGF	3	1.007 ± 0.017^##^
SM4 + TGF	3	0.648 ± 0.012^▲▲^
SM4 + TGF + SA-B	3	0.289 ± 0.022
SM4 + TGF + SB	3	0.281 ± 0.036
TGF + SA-B + SB	3	0.386 ± 0.024**
SM4 + TGF + SA-B + SB	3	0.222 ± 0.026^Δ^

The levels of Col. I protein were determined by ELISA kit as indicated. Results were expressed as mean ± SE of three independent experiments performed in triplicate. ^##^Significant difference versus Control, Negative control, and SRV4 (*n* = 3, *P* < 0.01); ^▲▲^Significant difference versus SRV4 and TGF-*β*1 (*n* = 3, *P* < 0.01); **Significant difference versus SRV4 (*n* = 3, *P* < 0.01); ^Δ^Significant difference versus SM4 + TGF + SA-B, SM4 + TGF + SB, and TGF + SA-B + SB (*n* = 3, *P* < 0.05).

## Appendix

ECM: Extracellular matrix
CCl_4_: Carbon tetrachloride
Col. I: Collagen type I
HSCs: Hepatic stellate cells
MFB: Myofibroblast-like cells
TGF-*β*1: Transforming growth factor-*β*1
*α*-SMA: *α*-smooth muscle actin
MAPK: Mitogen-activated protein kinase
SA-B: Salvianolic-acids B
SB: SB203580
PD: PD98059
SRV4: Smad4 RNAi
ERK: Extra cellular signal-regulated kinase
p38 MAPK: p38 mitogen-activated
RNAi: RNA interference
miRNA: MicroRNA
pri-miRNA: Primary transcript of miRNA
p-MEK: Phosphorylation of MEK
p-p38: Phosphorylation of p38
p-MKK3/6: Phosphorylation of MKK3/6.
